# Evaluation of the Psychosocial Impact of Dental Attractiveness Among the University of Khartoum Dental Students and Registrars

**DOI:** 10.1155/ijod/9110425

**Published:** 2026-10-14

**Authors:** Hiba Gafar A. Ahmed, Adil Osman Mageet

**Affiliations:** ^1^ Department of Orthodontics Faculty of Dentistry University of Khartoum Khartoum Sudan uofk.edu; ^2^ Department of Orthodontics Faculty of Dentistry Al Neelain University Khartoum Sudan neelain.edu.sd; ^3^ Department of Orthodontics Faculty of Dentistry University of Gezira Wad Madani Sudan uofg.edu.sd; ^4^ Department of Orthodontics Mediclinic Middle East Dubai UAE

**Keywords:** dental aesthetics, IOTN-AC, malocclusion, PIDAQ, psychosocial impact, University of Khartoum

## Abstract

**Background:**

Dental aesthetics play a significant role in psychosocial well‐being, especially among dental professionals who are both evaluators and subjects of aesthetic standards. In Sudan, limited data exist regarding the psychosocial impact of malocclusion among dental students and registrars.

**Aim:**

To assess the psychosocial impact of dental aesthetics among University of Khartoum dental students and registrars.

**Methods:**

A descriptive cross‐sectional study was conducted among 287 participants (188 dental students and 99 registrars) at the University of Khartoum, Faculty of Dentistry. Data were collected anonymously through a self‐administered electronic questionnaire using Google Forms. Participants were recruited using convenience sampling for undergraduate students and proportional cluster sampling for registrars. The questionnaire included demographic items, the self‐rated Index of Orthodontic Treatment Need‐Aesthetic Component (IOTN‐AC) and a modified 22‐item Psychosocial Impact of Dental Aesthetics Questionnaire (PIDAQ). Analyses included descriptive statistics, independent‐samples *t*‐tests, one‐way ANOVA, Pearson correlation, chi‐square tests and multiple linear regression using SPSS Version 22.

**Results:**

A total of 70.4% of participants selected IOTN‐AC photo 1 as most representative of their dentition, and 58.2% reported being satisfied with their dental appearance. There was a moderate positive correlation (*r* = 0.452, *p*  < 0.001) between self‐perceived IOTN‐AC severity and psychological‐impact scores. Females reported higher psychological‐impact scores (*p* = 0.008), while registrars reported lower scores than students (*p* = 0.015). Higher IOTN‐AC severity groups differed significantly in psychological‐impact scores (*F* = 4.567, *p* = 0.012).

**Conclusion:**

Self‐perceived dental aesthetic severity was associated with psychological impact among dental students and registrars. Psychological‐impact scores also differed by gender and participant role. These findings support consideration of psychosocial assessment in dental education and patient‐centred dental care.

## 1. Introduction

The dental appearance is among the first facial characteristics noticed during social interaction [1, 2]. Over the last three decades, it has become clear that deviations from prevailing aesthetic norms, whether objective (clinician‐rated) or subjective (patient‐rated), can compromise psychological well‐being, social confidence and even career prospects [3–5]. The World Health Organization’s definition of health as ‘complete physical, mental and social well‐being’ explicitly legitimises the study of such impacts [6]. Yet, in many low‐ and middle‐income countries (LMICs), including Sudan, research on the psychosocial consequences of malocclusion remains scarce [7].

Malocclusion is not a disease entity but a spectrum of morphological variations. Its functional consequences (masticatory inefficiency and periodontal risk) are sometimes less concerning to patients than the aesthetic stigma attached to irregular anterior teeth [8, 9]. Several epidemiological indices (Index of Orthodontic Treatment Need [IOTN], DAI and ICON) have been developed to quantify normative treatment need, but they were designed primarily for resource allocation rather than for capturing the lived experience of affected individuals [10–12]. Patient‐reported outcome measures (PROMs) such as the Oral Health Impact Profile (OHIP) and the shorter Psychosocial Impact of Dental Aesthetics Questionnaire (PIDAQ) have therefore gained prominence [13, 14]. PIDAQ in particular isolates four domains that resonate with orthodontic practice: dental self‐confidence, social impact, psychological impact and aesthetic concern [15].

Sub‐Saharan Africa displays unique sociocultural filters that modulate the judgement. Traditional practices (tooth filing and diastema appreciation) coexist with globalised media ideals of the ‘Hollywood smile’ [16, 17]. Economic barriers may further complicate access to orthodontic care and treatment uptake [18]. Understanding how these contextual factors shape the psychosocial burden of malocclusion is therefore essential for culturally congruent care.

Dental students and registrars occupy a distinctive vantage point: they possess the technical vocabulary to recognise malocclusion yet remain members of the same generational cohort as their future patients. Their self‐reflections may therefore act as an indicator of emerging societal attitudes. Previous work in Western settings has shown that dental education intensifies critical self‐scrutiny [19–21]. However, no study has examined whether advanced training buffers or exacerbates distress in an African context. Gender differences add another layer of complexity as women consistently report greater body‐focused anxiety across cultures [22–24].

Finally, the COVID‐19 pandemic has normalised virtual interaction, where the lower face is prominently displayed on screens, potentially increasing self‐awareness of the facial and dental appearance. Whether this phenomenon has amplified the psychosocial impact of malocclusion in Sudanese trainees has not been explored.

Recent evidence continues to support the value of combining self‐perceived orthodontic treatment need with patient‐reported measures of psychosocial impact. A 2026 study of 270 young adults administered both a culturally adapted version of the PIDAQ and the IOTN‐Aesthetic Component (IOTN‐AC) and found that participants with greater IOTN‐AC severity had significantly higher PIDAQ scores (*p*  < 0.001) [25]. Similarly, a 2026 study of 306 orthodontic patients using PIDAQ, IOTN‐AC and clinician‐assessed IOTN‐DHC found that psychosocial impact was associated with older age, female gender, perfectionism and greater self‐perceived aesthetic need, whereas clinician‐assessed treatment need was not significantly associated with PIDAQ scores [26].

Therefore, this study aimed to assess the psychosocial impact of dental aesthetics among dental students and registrars at the University of Khartoum and to examine its association with self‐perceived IOTN‐AC severity, gender and participant role.

## 2. Materials and Methods

This descriptive cross‐sectional study was carried out at the University of Khartoum, Faculty of Dentistry, from January to April 2025. A total of 287 participants (188 dental students and 99 registrars) were included.

Undergraduate dental students and registered postgraduate registrars were invited to participate. The sample size for undergraduate dental students was calculated using the standard power analysis assumptions stated in the thesis: *α* = 0.05 (*Z*1−*α*/2 = 1.96), statistical power = 90% (*Z*1−*β* = 1.28) and effect size (Δ) = 0.24. Using the formula *N* = [(*Z*1−*α*/2 + *Z*1−*β*)^2^/Δ^2^] + [*Z*1−*α*/2^2^/2], the calculated target was 184 students [27]. For registrars, 92 participants were targeted using proportional cluster sampling based on the departmental registrar population (*N* = 114). The planned sample was therefore 276 participants. The final analysed sample comprised 287 participants: 188 students (184 planned plus 4 additional eligible respondents) and 99 registrars (92 planned plus 7 additional eligible respondents).

Inclusion criteria were Sudanese nationality, age 16 years or older, willingness to participate and absence of systemic or craniofacial conditions affecting the dental appearance. Exclusion criteria were craniofacial syndromes or anomalies, anterior lesions due to caries, trauma, fluorosis, or hypoplasia and a history of prior orthodontic or cosmetic dental treatment because such treatment may alter self‐perceived dental aesthetics independently of the underlying malocclusion.

The questionnaire was distributed electronically via Google Forms to eligible undergraduate students and postgraduate registrars at the University of Khartoum, Faculty of Dentistry, and was completed independently online. Dental students were recruited using convenience sampling, while registrars were recruited using proportional cluster sampling. The self‐administered questionnaire comprised demographic information (age, gender and academic level), the self‐rated IOTN‐AC and a modified 22‐item PIDAQ. The questionnaire used for data collection is provided in Appendix I. The modified PIDAQ retained items 1–22 of the original 23‐item instrument; the original final item concerning viewing one’s teeth in a video was not included. The PIDAQ items were scored on a five‐point Likert scale from 0 (strongly disagree) to 4 (strongly agree), covering dental self‐confidence (items 1–6), social impact (items 7–14), psychological impact (items 15–20) and aesthetic concern (items 21–22). The IOTN‐AC photographic scale and assessment material are provided in Appendix III.

Ethical approval for this study was granted by the Research Ethics Committee, Faculty of Dentistry, University of Khartoum. The study was conducted in accordance with the ethical principles of the World Medical Association Declaration of Helsinki. Written informed consent was obtained from all participants before questionnaire completion. The participant informed consent form is provided in Appendix II. Participants were informed of the study purpose, procedures, confidentiality safeguards and their right to request withdrawal of their data at any time before data analysis without providing a reason. Data were collected anonymously.

All data were transferred to an Excel sheet and analysed using SPSS Version 22 (IBM Corp., USA). Descriptive statistics summarised the frequencies, percentages, means and standard deviations. Independent‐samples *t*‐tests were used for two‐group comparisons, one‐way ANOVA for comparisons across IOTN‐AC severity groups, Pearson correlation to examine the relationship between self‐rated IOTN‐AC and psychological‐impact scores, chi‐square tests for item‐response distributions, and multiple linear regression to examine predictors of the psychological impact. Composite PIDAQ subscale scores were treated as approximately continuous variables. Normality and equality of variance were assessed by visual inspection and Levene’s test. All tests were two‐tailed, with *p*  < 0.05 considered statistically significant.

## 3. Results

### 3.1. Demographic Summary

Age distribution: Most respondents (58.9%, *n* = 169) were aged 16–25 years. Participants aged 26–35 years accounted for 35.5% (*n* = 102), while 5.2% (*n* = 15) were aged 36–45 years and 0.3% (*n* = 1) were aged 46–55 years (Table 1).

**Table 1:** Demographic characteristics of study participants.

Category	Subgroup	Frequency	Percentage (%)
Age	16–25 years	169	58.9
Age	26–35 years	102	35.5
Age	36–45 years	15	5.2
Age	46–55 years	1	0.3
Gender	Female	216	75.3
Gender	Male	71	24.7
Role	Registrar	99	34.5
Role	5th‐grade students	66	23.0
Role	4th‐grade students	73	25.4
Role	3rd‐grade students	23	8.0
Role	2nd‐grade students	18	6.3
Role	1st‐grade students	8	2.8

Gender demographics: Females represented the largest demographic segment at 75.3% (*n* = 216), followed by males at 24.7% (*n* = 71) (Table 1).

Grade/role distribution: Registrars formed the largest group (34.5%, *n* = 99), with 4th‐grade students as the next largest group (25.4%, *n* = 73). Fifth‐grade students made up 23.0% (*n* = 66), while 3rd‐grade (8.0%, *n* = 23), 2nd‐grade (6.3%, *n* = 18) and 1st‐grade students (2.8%, *n* = 8) constituted smaller proportions (Table 1).

### 3.2. PIDAQ

The distribution of Likert scale responses for the PIDAQ items is presented in Table 2.

**Table 2:** Likert‐scale response distribution.

Number	Statement	Strongly agree	Agree	Neutral	Disagree	Strongly disagree
1	I am proud of my teeth	22.3	33.4	34.8	9.1	0.7
2	I am satisfied with my teeth appearance	28.2	30.0	30.7	10.1	1.4
3	I am pleased seeing my teeth in the mirror	23.7	34.1	26.8	13.6	1.7
4	I find my tooth position very nice	24.7	29.6	25.4	17.8	2.4
5	I like to show my teeth when smiling	26.8	32.8	22.3	15.3	3.1
6	My teeth are attractive to others	16.7	26.8	39.7	14.3	2.4
7	I hold myself back when smiling	3.1	15.0	17.4	37.3	27.2
8	Concerned about others’ opinions	1.7	17.8	18.8	31.7	30.0
9	Fear offensive remarks about teeth	1.4	11.1	14.6	38.3	34.5
10	Inhibited in social contacts	1.0	3.1	9.1	36.9	49.8
11	Hide teeth with hand	2.8	10.8	10.1	30.7	45.6
12	Think people stare at teeth	2.1	9.1	28.2	33.8	26.8
13	Irritated by jokes about teeth	1.4	13.6	17.4	38.7	28.2
14	Worry about opposite sex’s opinion	1.0	9.4	13.9	35.9	39.7
15	Unhappy about teeth appearance	1.0	25.4	17.1	29.3	27.2
16	Envy others’ teeth	3.8	16.4	27.5	23.3	28.9
17	Others have nicer teeth	2.8	9.4	28.2	41.5	18.5
18	Feel bad about teeth appearance	0.3	8.4	19.5	35.2	36.9
19	Wish teeth looked better	12.5	38.0	22.3	15.7	12.5
20	Distressed by others’ teeth	0.3	4.5	23.0	39.0	33.4
21	Dislike seeing teeth in the mirror	0.3	6.3	17.4	40.8	35.2
22	Dislike seeing teeth in photos	2.1	12.2	17.4	36.6	31.7

*Note:* Likert scale scoring: 0 = strongly disagree, 1 = disagree, 2 = neutral, 3 = agree, and 4 = strongly agree.

#### 3.2.1. Dental Self‐Confidence Domain (Items 1–6)

More than half of the participants expressed positive perceptions of their dental aesthetics. Specifically, 55.7% (22.3% strongly agree and 33.4% agree) reported being proud of their teeth, while 34.8% remained neutral. Similarly, 58.2% (28.2% strongly agree and 30.0% agree) were satisfied with their dental appearance and 57.8% (23.7% strongly agree and 34.1% agree) felt pleased when seeing their teeth in the mirror. Regarding tooth position, 54.3% (24.7% strongly agree and 29.6% agree) considered their tooth alignment to be aesthetically pleasing. A total of 59.6% (26.8% strongly agree and 32.8% agree) reported feeling comfortable showing their teeth when smiling. However, perceptions of attractiveness to others were less pronounced, with 43.5% (16.7% strongly agree and 26.8% agree) agreeing that their teeth were attractive, while 39.7% selected a neutral response.

#### 3.2.2. Social Impact Domain (Items 7–14)

Most respondents reported minimal negative social impact related to their dental appearance. The majority (64.5%; 37.3% disagree and 27.2% strongly disagree) indicated that they did not hold themselves back when smiling. Similarly, 61.7% (31.7% disagree and 30.0% strongly disagree) were not concerned about others’ opinions, and 72.8% (38.3% disagree and 34.5% strongly disagree) did not fear offensive remarks about their teeth. Social inhibition due to dental appearance was uncommon, with 86.7% (36.9% disagree and 49.8% strongly disagree) denying feeling inhibited in social contacts. Additionally, 76.3% (30.7% disagree and 45.6% strongly disagree) reported that they did not hide their teeth with their hands. Perceived scrutiny from others was also low, as 60.6% (33.8% disagree and 26.8% strongly disagree) disagreed with the statement that people stare at their teeth. Furthermore, 66.9% (38.7% disagree and 28.2% strongly disagree) were not irritated by jokes about their teeth and 75.6% (35.9% disagree and 39.7% strongly disagree) did not worry about the opposite sex’s opinion regarding their teeth.

#### 3.2.3. Psychological Impact Domain (Items 15–20)

Responses indicated generally low levels of psychological distress. More than half of participants (56.5%; 29.3% disagree and 27.2% strongly disagree) did not report being unhappy with their dental appearance. Envy toward others’ teeth was also uncommon, with 52.2% (23.3% disagree and 28.9% strongly disagree) expressing disagreement. Likewise, 60.0% (41.5% disagree and 18.5% strongly disagree) did not believe that others had nicer teeth. Feelings of discomfort when thinking about their teeth were minimal, with 72.1% (35.2% disagree and 36.9% strongly disagree) denying feeling bad about their dental appearance. In addition, 50.5% (38.0% agree and 12.5% strongly agree) wished that their teeth looked better, whereas 72.4% (39.0% disagree and 33.4% strongly disagree) were not distressed by other people’s teeth.

#### 3.2.4. Aesthetic Concern Domain (Items 21–22)

Most participants reported comfort when seeing their teeth in mirrors and photographs. Specifically, 76.0% (40.8% disagree and 35.2% strongly disagree) disagreed with disliking their teeth in the mirror, while 68.3% (36.6% disagree and 31.7% strongly disagree) disagreed with disliking their teeth in photographs.

### 3.3. IOTN‐AC

Most respondents (70.4%) selected photo 1 as the most representative of their own dentition, indicating a strong self‐perception of good dental appearance (Table 3). A smaller group (13.2%) chose photo 2, while 8.0% selected photo 3; the remaining 8.4% were distributed across photos 4–10, indicating varied perceptions of personal dental aesthetics.

**Table 3:** IOTN‐AC response distribution.

Aesthetic component photo	Frequency	Percent
1	202	70.4
2	38	13.2
3	23	8.0
4	16	5.6
5	2	0.7
6	2	0.7
7	1	0.3
8	2	0.7
10	1	0.3
Total	287	100.0

The large majority of participants (97.2%) self‐rated IOTN‐AC grades 1–4 (no or slight aesthetic impairment), while only 1.7% self‐rated grades 5–7 (moderate impairment) and 1.0% self‐rated grades 8–10 (severe impairment) (Table 3).

Mean PIDAQ subscale scores are presented in Table 4. Key inferential statistics are summarised in Table 5. Psychological impact was significantly correlated with self‐perceived IOTN‐AC severity (*r* = 0.452, *p* < 0.001). Significant differences were observed by gender (*t* = −2.663, *p* = 0.008) and participant role (*t* = −2.456, *p* = 0.015) and across IOTN‐AC severity groups (*F* = 4.567, *p* = 0.012). Multiple regression identified grade level and registrar status as significant predictors of psychological impact (*R* ^2^ = 0.125, *p* < 0.001) (Table 5).

**Table 4:** Mean PIDAQ subscale scores (*n* = 287).

PIDAQ subscale	Mean ± SD
Dental self‐confidence	2.8 ± 0.8
Social impact	1.9 ± 0.9
Psychological impact	3.1 ± 1.0
Aesthetic concern	2.4 ± 1.1

**Table 5:** Associations between psychological impact and key variables (*n* = 287).

Variable	Statistic	Value	*p*‐Value
Correlation: IOTN‐AC vs. psychological impact	*r*	0.452	<0.001
Gender difference (female vs. male)	*t*	−2.663	0.008
Role difference (registrar vs. student)	*t*	−2.456	0.015
IOTN‐AC severity groups	*F*	4.567	0.012
Regression: grade level	*β*	−0.25	<0.001
Regression: registrar status	*β*	−0.18	<0.001
Regression model	*R* ^2^	0.125	<0.001

## 4. Discussion

The present study demonstrates a significant association between self‐perceived IOTN‐AC severity and the psychological impact among dental students and registrars. The observed moderate correlation indicates that higher self‐perceived dental irregularity was associated with higher psychological‐impact scores. Because the IOTN‐AC was self‐rated, these findings should be interpreted as associations between perceived dental aesthetics and psychosocial impact rather than as evidence of objectively measured malocclusion severity.

The social significance of dentofacial appearance has been well established, with early evidence demonstrating that variations in dental aesthetics exert a measurable influence on perceived attractiveness and social judgement [1]. The current findings corroborate this concept as participants consistently identified dental appearance as a key determinant of self‐perception and social confidence. This supports the notion that aesthetic concerns are intrinsically linked to psychosocial well‐being and cannot be regarded as purely cosmetic or secondary considerations.

Contemporary evidence further substantiates the central role of subjective perception in shaping psychosocial outcomes [3]. It has been demonstrated that self‐perceived orthodontic treatment need may exert a stronger influence on psychological well‐being than clinician‐assessed malocclusion severity. The present findings are consistent with this distinction as psychosocial responses may vary among individuals with similar perceived dental characteristics.

This positive correlation between self‐perceived malocclusion severity (IOTN‐AC) and psychological impact aligns with recent work confirming associations between PIDAQ scores and malocclusion‐related aesthetic outcomes [28]. Similarly, a 2026 study of dental students found that self‐perceived aesthetic treatment need was significantly associated with the psychosocial impact of dental appearance [29]. In the present study, the IOTN‐AC was self‐rated and therefore should not be interpreted as an objective clinical assessment of malocclusion severity.

This finding is further supported by additional recent evidence. A 2026 study of 270 young adults similarly found that greater IOTN‐AC severity was associated with significantly higher PIDAQ scores (*p* < 0.001) [25], consistent with the moderate positive correlation observed in the present study (*r* = 0.452, *p* < 0.001). Further support comes from a 2026 study of 306 orthodontic patients using PIDAQ, IOTN‐AC and clinician‐assessed IOTN‐DHC, which found that psychosocial impact was associated with older age, female gender, perfectionism and greater self‐perceived aesthetic need, whereas clinician‐assessed treatment need was not significantly associated with PIDAQ scores [26]. These findings reinforce the distinction between perceived aesthetic need and professionally assessed orthodontic need and support the relevance of subjective dental aesthetic perception to psychosocial well‐being observed in the present study.

Moreover, differences in the psychological impact between students and registrars may reflect differences in academic level, clinical exposure, or professional experience. In the present study, undergraduate students reported greater levels of psychological distress than registrars. However, because the study was cross‐sectional, these differences should be interpreted as associations rather than evidence that clinical training directly reduces psychological distress.

The role of self‐perceived treatment need in psychosocial responses is noteworthy [3]. The present findings suggest that subjective perception is associated with emotional response, but the cross‐sectional design does not allow mediation or causal relationships to be established. A patient‐centred approach should therefore consider both clinical findings and individual psychosocial context.

Consistent with previous research, malocclusion in young adults is associated with diminished self‐esteem and increased social avoidance behaviours [4]. The current results align closely with this evidence, particularly among student participants, further supporting the notion that dental aesthetics play a significant role in shaping psychosocial well‐being during early adulthood.

The finding that female participants reported significantly higher psychological impact is consistent with several recent studies. Research among university students in Serbia similarly examined the psychosocial effects of dental aesthetics and self‐esteem [30]. However, this relationship is not universally replicated: a 2025 study among Spanish education centre students found no significant association between psychosocial impact and gender, despite significant gender differences in self‐esteem [31], suggesting that cultural and contextual factors may moderate this relationship rather than a fixed biological effect.

Emerging evidence also highlights the role of socioeconomic and contextual factors in shaping aesthetic satisfaction and treatment expectations [9]. The present study supports this perspective as psychosocial impact was influenced not only by perceived severity but also by broader individual and environmental factors.

Despite the widespread use of orthodontic indices, clinical findings do not always align with patient‐perceived needs [10]. The present study demonstrates variability in the psychological impact among participants reporting dental aesthetic characteristics, highlighting the value of incorporating validated psychosocial instruments such as PIDAQ alongside orthodontic assessment.

Clinical training has been shown to influence aesthetic judgement, with more experienced individuals demonstrating a more analytical and less emotionally reactive approach to the dental appearance [19]. This is consistent with the lower psychological‐impact scores reported by registrars in the present study. The lower distress reported by registrars compared with that of students may reflect differences in advancing dental education and clinical exposure, a pattern echoed in a Romanian study of dental medicine students, where psychosocial impact and self‐esteem varied by academic level and prior dental knowledge [32]. Nevertheless, the cross‐sectional design prevents causal conclusions about whether clinical training itself reduces psychosocial vulnerability.

Furthermore, disparities in aesthetic perception between dental professionals, students, and laypersons have been well documented [20]. These differences reinforce the concept that clinical knowledge and exposure can alter perceptual frameworks, potentially reducing psychosocial sensitivity to malocclusion.

Cultural and perceptual variability further complicates the interpretation of dental aesthetics, as standards of attractiveness are not universally defined [23]. The present findings reflect this diversity, with differing psychosocial responses suggesting that aesthetic perception is shaped by both individual and sociocultural influences.

In addition to clinical and perceptual factors, individual psychological traits such as self‐esteem and perfectionism contribute to variability in psychosocial outcomes [24]. The observed heterogeneity in PIDAQ responses among participants with similar clinical profiles suggests that intrinsic psychological predispositions influence sensitivity to dental aesthetics. This highlights the multifactorial nature of the psychosocial impact and the need for a more holistic assessment framework.

Overall, the present findings emphasise that the psychosocial impact of malocclusion is shaped by a complex interplay between perceived aesthetic concerns, individual psychological factors and clinical experience. These findings underscore the importance of incorporating psychosocial assessment into routine dental care to better identify students and clinicians who may benefit from targeted support.

This study has several limitations. It was conducted at a single institution, which may limit generalisability. The use of convenience sampling for undergraduate students may introduce selection bias, while the proportional cluster approach used for registrars may limit comparability between the two recruitment groups. The cross‐sectional design does not permit a causal inference. The questionnaire was self‐administered and may be subject to response bias, and IOTN‐AC was self‐rated rather than clinician‐assessed, limiting conclusions about objectively assessed malocclusion. In addition, the study used a modified 22‐item PIDAQ structure, which should be considered when comparing scores with those of studies using the original instrument. Nevertheless, the study provides baseline evidence on the psychosocial impact of dental aesthetics among Sudanese dental students and registrars.

## 5. Conclusion

Self‐perceived dental aesthetic severity was associated with a psychological impact among dental students and registrars. Psychological‐impact scores also differed by gender and participant role, while higher IOTN‐AC severity was associated with greater psychological impact. Because the study was cross‐sectional and IOTN‐AC was self‐rated, these findings should be interpreted as associations. Future educational initiatives may benefit from incorporating validated psychosocial assessment tools such as the PIDAQ into patient‐centred dental education and care.

## Author Contributions

Conceptualisation, methodology, investigation, data curation, formal analysis, writing – original draft: Hiba Gafar A. Ahmed. Formal analysis, writing – review and editing: Hiba Gafar A. Ahmed and Adil Osman Mageet. Supervision: Adil Osman Mageet.

## Funding

This study did not receive any specific funding.

## Disclosure

All authors have read and approved the final version of the manuscript. Hiba Gafar A. Ahmed had full access to all of the data in this study and takes complete responsibility for the integrity of the data and the accuracy of the data analysis. Adil Osman Mageet, the corresponding author, is responsible for communication with the journal on behalf of all authors.

## Ethics Statement

Ethical approval for this study was granted by the Research Ethics Committee, Faculty of Dentistry, University of Khartoum. The study was conducted in accordance with the ethical principles of the World Medical Association Declaration of Helsinki. All procedures were conducted in accordance with the approved research protocol and applicable ethical requirements.

## Consent

Written informed consent was obtained from all participants prior to questionnaire completion, following an explanation of the study’s purpose, procedures and confidentiality safeguards. Participants were informed of their right to withdraw their data from the study at any point prior to data analysis without providing a reason.

## Conflicts of Interest

The authors declare no conflicts of interest.

## Supporting Information

Additional supporting information can be found online in the Supporting Information section.

## Supporting information


**Supporting Information** The following Supporting Information is available for this article as a unified document in the Supporting Information section: Appendix I: Questionnaire used for data collection, including demographic items and the modified 22‐item PIDAQ. Appendix II: Participant informed consent form. Appendix III: Index of Orthodontic Treatment Need‐Aesthetic Component (IOTN‐AC) photographic scale. Appendix IV: Institutional ethics committee approval letter. Appendix V: Completed STROBE checklist for cross‐sectional studies.

## Data Availability

Data are available from the corresponding author upon reasonable request, subject to applicable ethical and institutional restrictions.
