# Supplementary material for: Evaluation of the Psychosocial Impact of Dental Attractiveness Among the University of Khartoum Dental Students and Registrars
Source: Int J Dent. 2026 Oct 14;2026:9110425. doi: 10.1155/ijod/9110425 (PMC13669822; doi:10.1155/ijod/9110425)
Supplement: Supplementary file 1 [file IJOD-2026-9110425-s001.pdf]

# SUPPLEMENTARY MATERIALS

Study Title: Evaluation of the Psychosocial Impact of Dental Attractiveness Among the University of Khartoum Dental Students and Registrars

Authors: Hiba Gafar A. Ahmed, Adil Osman Mageet

## Appendix I: Study Questionnaire

### Section A: Demographics

Age: __________

Gender: [ ] Female   [ ] Male

Participant Role: [ ] Undergraduate Student ( Year: ___ )   [ ] Postgraduate Registrar

### Section B: Modified Psychosocial Impact of Dental Aesthetics Questionnaire (PIDAQ)

Please rate the following statements on a scale from 0 to 4:
(0 = Strongly Disagree, 1 = Disagree, 2 = Neutral, 3 = Agree, 4 = Strongly Agree)

1. I am proud of my teeth
2. I am satisfied with the appearance of my teeth
3. I am pleased when I see my teeth in the mirror
4. I find my tooth position to be very nice
5. I like to show my teeth when I smile
6. My teeth are attractive to others
7. I hold myself back when I smile so my teeth do not show so much
8. I am sometimes concerned about what people might think about my teeth
9. I am afraid other people could make offensive remarks about my teeth
10. I am somewhat inhibited in social contacts because of my teeth
11. I sometimes hold my hand in front of my mouth to hide my teeth
12. Sometimes I think people are staring at my teeth
13. Remarks about my teeth irritate me even when they are meant jokingly
14. I sometimes worry about what members of the opposite sex think about my teeth
15. Sometimes I am somewhat unhappy about the appearance of my teeth
16. I envy the nice teeth of other people
17. I think most people I know have nicer teeth than I do
18. I feel bad when I think about what my teeth look like
19. I wish my teeth looked better
20. I am somewhat distressed when I see other people's teeth

21. I do not like to see my teeth in the mirror

22. I do not like to see my teeth in photographs

### Section C: Index of Orthodontic Treatment Need – Aesthetic Component (IOTN-AC)

According to the ten colour photographs of anterior teeth shown to you in Appendix III, which photograph do you feel resembles your dentition most closely?

Selected Photo Number (1–10): __________

## Appendix II: Participant Informed Consent Form

Study Title: Evaluation of the Psychosocial Impact of Dental Attractiveness Among the University of Khartoum Dental Students and Registrars

Principal Investigator: Hiba Gafar A. Ahmed, Registrar, Department of Orthodontics, Faculty of Dentistry, University of Khartoum

**Purpose:** This study aims to assess the psychosocial impact of dental aesthetics among University of Khartoum dental students and registrars.

**Procedures:** Participation involves filling out an anonymous electronic questionnaire containing demographic items, 22 PIDAQ questions, and a single self-rated IOTN-AC assessment.

**Benefits:** Data will help identify the psychosocial impact of malocclusion to support patient-centred orthodontic care.

**Time:** Completing the questionnaire takes approximately 5–10 minutes.

**Confidentiality & Voluntariness:** Participation is completely voluntary. All responses are anonymous. You may withdraw your data at any time prior to data analysis without penalty or explanation.

**Contact:** hibagafar@neelain.edu.sd

## Appendix III: IOTN-AC Photographic Scale

(Insert high-resolution image file Figure_A1_IOTN_AC.png showing photos 1–10 here)

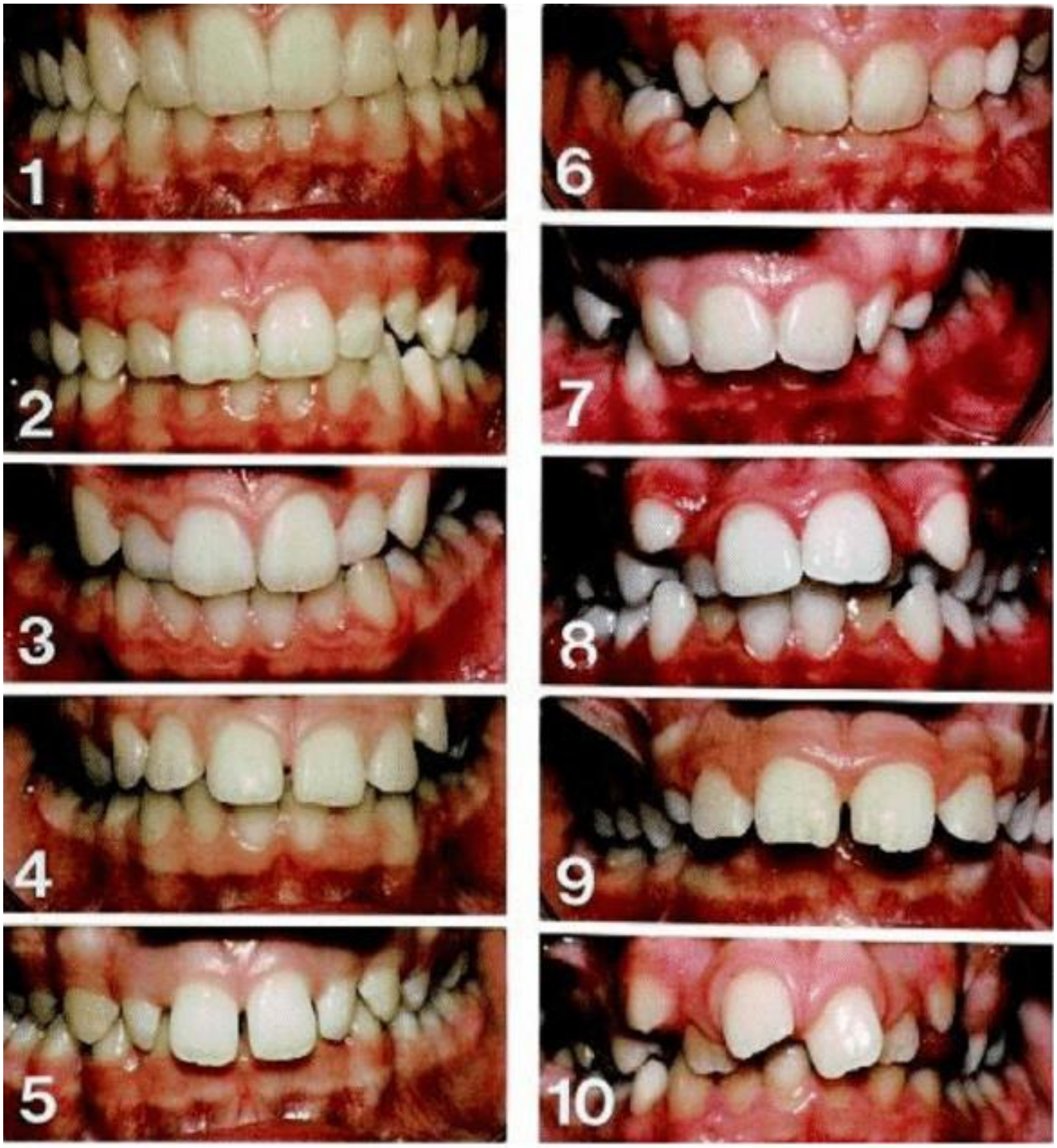

## Appendix IV: Ethical Approval Documentation

**Ethical Approval Letter – Research Ethics Committee, Faculty of Dentistry, University of Khartoum**

University of Khartoum — Faculty of Dentistry

Date: 14 February 2025

**ETHICAL APPROVAL STATEMENT**

Date: 14 February 2025

# ETHICAL APPROVAL STATEMENT

To Whom It May Concern

**Study Title:** Psychosocial Impact of Dental Aesthetics among Sudanese Dental Students and Registrars: A Cross-Sectional Study
**Principal Investigator:** Hiba Gafar Abdelkhalig Ahmed

This is to certify that the above-mentioned research project was reviewed and approved by the Research Ethics Committee, Faculty of Dentistry, University of Khartoum, from an ethical perspective.

The study was conducted in accordance with the ethical principles of the World Medical Association Declaration of Helsinki. Written informed consent was obtained from all participants prior to their participation in the study. All research procedures were conducted in accordance with the approved research protocol and applicable ethical requirements.

This statement is issued upon request for documentation of the ethical approval of the above-mentioned research project.

On behalf of the Faculty of Dentistry, University of Khartoum,

Dean, Faculty of Dentistry
University of Khartoum

Name: Abbas Gareeb alla

Signature: ____________

Official Stamp: __________

University of Khartoum جامعة الخرطوم Dean office مكتب العميد كلية طب الأسنان Faculty of Dentistry

## Appendix V: STROBE Statement Checklist

## STROBE Statement — Checklist of items that should be included in reports of cross-sectional studies

Manuscript ID: 9110425 — Evaluation of the Psychosocial Impact of Dental Attractiveness Among the University of Khartoum Dental Students and Registrars

| Item | Recommendation | Reported on (section/paragraph) | Status |
|---|---|---|---|
| 1a | Indicate the study's design in the title or abstract | Abstract — "descriptive cross-sectional study" | ✓ |
| 1b | Provide an informative and balanced summary in the abstract | Abstract (Aim, Methods, Results, Conclusion) | ✓ |
| 2 | Explain the scientific background and rationale for the investigation | Introduction, paragraphs 1–5 | ✓ |
| 3 | State specific objectives, including any prespecified hypotheses | Introduction, final paragraph; Abstract – Aim | ✓ |
| 4 | Present key elements of study design early in the paper | Methods, paragraph 1 | ✓ |
| 5 | Describe the setting, locations, and relevant dates of recruitment, exposure, follow-up, and data collection | Methods, paragraph 1 (University of Khartoum, Jan–Apr 2025) | ✓ |
| 6a | Give eligibility criteria, sources and methods of selection of participants | Methods, paragraphs 2–3 (inclusion/exclusion criteria, sample size) | ✓ |
| 7 | Clearly define all outcomes, exposures, predictors, potential confounders, and effect modifiers | Methods, paragraph 4 (IOTN-AC, PIDAQ subscales) | ✓ |
| 8 | For each variable of interest, give sources of data and details of methods of assessment | Methods, paragraph 4 (questionnaire instruments) | ✓ |
| 9 | Describe any efforts to address potential sources of bias | Methods, paragraphs 2–3 (exclusion of prior orthodontic treatment) | ✓ |
| 10 | Explain how the study size was arrived at | Methods, paragraph 2 (sample size calculation) | ✓ |

| | | | |
|---|---|---|---|
| 11 | Explain how quantitative variables were handled in the analyses | Methods, paragraph 5 (descriptive/inferential statistics) | ✓ |
| 12a | Describe all statistical methods, including those used to control for confounding | Methods, paragraph 5 | ✓ |
| 12b | Describe any methods used to examine subgroups and interactions | Methods, paragraph 5 (t-test, ANOVA, regression by gender/role) | ✓ |
| 12c | Explain how missing data were addressed | Not applicable — no missing data (electronic form required all fields) | ✓ |
| 12d | If applicable, describe analytical methods taking account of sampling strategy | Methods, paragraph 2 (sampling strategy) | ✓ |
| 12e | Describe any sensitivity analyses | Not applicable | N/A |
| 13a | Report numbers of individuals at each stage of the study | Results, Table 1 (n=287; 188 students, 99 registrars) | ✓ |
| 13b | Give reasons for non-participation at each stage | Not separately reported — to be added if non-response data available | ⚠ partial |
| 13c | Consider use of a flow diagram | Not included — optional for this design | N/A |
| 14a | Give characteristics of study participants and information on exposures and potential confounders | Results, Table 1 (demographic summary) | ✓ |
| 14b | Indicate number of participants with missing data for each variable of interest | Not applicable — no missing data | ✓ |
| 15 | Report numbers of outcome events or summary measures | Results, Tables 2–5 (PIDAQ/IOTN-AC distributions and summary statistics) | ✓ |
| 16a | Give unadjusted estimates and, if applicable, confounder-adjusted estimates and their precision | Results, Table 4 and Table 5 (means ± SD, t/F statistics, p-values) | ✓ |
| 16b | Report category boundaries when continuous variables were categorized | Results, Table 3 (IOTN-AC grade groupings 1–4, 5–7, 8–10) | ✓ |

| | | | |
|---|---|---|---|
| 16c | If relevant, translate relative risk into absolute risk | Not applicable — no risk ratios calculated | N/A |
| 17 | Report other analyses done, e.g. subgroup analyses, interactions, sensitivity analyses | Results, Table 5 (regression analysis with grade level, registrar status) | ✓ |
| 18 | Summarise key results with reference to study objectives | Discussion, paragraph 1 | ✓ |
| 19 | Discuss limitations of the study, addressing sources of potential bias or imprecision | Discussion, final paragraph (single-institution setting, cross-sectional design, self-report bias) | ✓ Discussion – Limitations paragraph |
| 20 | Give a cautious overall interpretation considering objectives, limitations, multiplicity of analyses, and similar studies | Discussion, paragraphs throughout (compared against refs 1–30) | ✓ |
| 21 | Discuss the generalisability of the study results | Discussion, final paragraph (single-institution generalisability addressed) | ✓ Discussion – Generalisability discussed |
| 22 | Give the source of funding and the role of the funders | Funding statement (no specific funding received) | ✓ |

*Item 13b (reasons for non-participation) is marked partial since non-response data were not separately tracked during Google Forms distribution. All other items, including Items 19 (limitations) and 21 (generalisability), are fully addressed following the addition of a dedicated limitations paragraph at the end of the Discussion.*
